# Histopathological Picture of Lung Organs Towards Combination of Java Cardamom Seed Extract and Turmeric Rhizome as Anti-Colibacillosis in Broiler Chickens

**DOI:** 10.3390/vetsci12080726

**Published:** 2025-07-31

**Authors:** Tyagita Hartady, Mohammad Ghozali, Charles Parsonodihardjo

**Affiliations:** 1 Department of Biomedical Science, Faculty of Medicine, Universitas Padjadjaran, Sumedang 45363, Indonesia; moh.ghozali@unpad.ac.id; 2 Veterinary Medicine Study Program, Faculty of Medicine, Universitas Padjadjaran, Sumedang 45363, Indonesia; charles20001@mail.unpad.ac.id

**Keywords:** *amomum compactum*, antibiotics, antimicrobial resistance, *curcumin*, *cineol*

## Abstract

Colibacillosis is a poultry disease caused by the pathogenic bacterium *Escherichia coli* (*E. coli*). This study is an experimental cross-sectional study using herbal-based test materials from Javanese cardamom and turmeric rhizome as treatments to replace the role of antibiotics that experience drug resistance in several types of bacteria. A total of 32 samples were utilized in this study, separated into two control groups and six treatment groups. The analysis was carried out by an histopathological examination of the lung organs using H&E and ImageJ staining to calculate the area of the slide image. The data results were analyzed statistically with one-way ANOVA method and qualitatively. The outcome of the statistical test showed that the differences were not statistically significant *p* value = 0.922 [*p* > 0.05] in all groups, and findings from qualitative histopathology showed morphological differences in the alveoli, parabronchi, and vasculature in the lung organs.

## Simple Summary

Colibacillosis is a poultry disease that significantly affects poultry industry production. *Escherichia coli* infection in chicken causes high mortality rates and great losses for farmers and companies. Antibiotics are commonly used for treatment, but high uses of antibiotics cause resistance by the pathogen. In this research, we looked at herbal treatment approaches along with the natural wealth of nature, in particular, Javanese cardamom and turmeric rhizome extract, to provide bactericidal substances to treat bacterial infections. We use Imagej 1.54p to provide better quantitative measurements and we do qualitative analysis for overall pathological lesions on the tissue.

## 1. Introduction

The broiler farming industry continues to face significant challenges due to diseases that negatively impact productivity and meat quality. Among these, colibacillosis remains one of the most prevalent & economically damaging conditions affecting broiler chickens [1]. Colibacilosis is primarily induced by Avian Pathogenic *Escherichia coli* (APEC), a pathogen widely distributed in poultry production systems, particularly in intensive farming environments. APEC infections can occur across all age groups and are a major concern globally [2]. Colibacillosis is a systemic and localized infection characterized by multi-organ lesions, including pericarditis, peritonitis, airsacculitis, salpingitis, synovitis, yolk sac infections, and head swelling syndrome [3]. The condition primarily affects broilers and laying hens during the age range of three to five weeks [1]. Despite commonly occurring as a secondary infection after diseases like infectious bronchitis, infectious bursal disease, or peritonitis, colibacillosis remains a preeminent cause of ill health & death in commercial poultry production [4].

*Escherichia coli* is a prevalent microbe that naturally populate at the gastrointestinal and respiratory systems of chickens. However, certain strains, classified as Avian Pathogenic *Escherichia coli* (APEC), can induce severe disease in chickens, particularly under stress or when their immune defenses are compromised. These pathogenic strains are often found in the intestines, which act as the main reservoir [5]. APEC can enter the bloodstream and cause colibacillosis, a disease that damages several vital organs, including the lungs [6]. The lungs are important for breathing, heat regulation, and removing toxins, so any damage can greatly affect the bird’s health and survival.

Colibacillosis often spreading to the respiratory tract, especially by entering the bloodstream, which is the most effective route of infection compared to oral or nasal route/pathway [7]. Infection typically begins when the lining of the respiratory tract is damaged, allowing bacteria to more easily invade [8]. Once infected, the lungs may show signs of swelling, bleeding, and cell death [6], and the disease can progress to chronic respiratory problems and air sacculitis [9]. Microscopic changes in the lungs may include congestion, hemorrhage, and the buildup of immune cells in the air spaces [3]. Colibacillosis causes both localized and widespread damage, including pericarditis, peritonitis, salpingitis, synovitis, yolk sac infections, and head swelling syndrome. It is most common in broilers and laying hens aged 3 to 5 weeks [1]. Although often triggered by other diseases like infectious bronchitis or bursal disease, colibacillosis can lead to high mortality rates in poultry flocks [4]. Colibacillosis is commonly treated using broad-spectrum antibiotics [10]. The escalating antibiotic resistance in pathogenic *Escherichia coli* strains poses a critical challenge for treatment efficacy [11].

In broiler farms across West Java, high levels of Antibiotic resistance has been identified in *Escherichia coli* strains isolates, highlighting the widespread nature of the issue [12]. APEC strains have shown alarming resistance rates to several antibiotics, including High levels of AMR in *Escherichia coli* were recorded for ampicillin and tetracycline (100%), followed by amoxicillin/clavulanic acid (16.25%), nalidixic acid (95%), ofloxacin (93.75%), doxycycline (91.87%), ciprofloxacin (87.50%), gentamicin (32.50%), trimethoprim/sulfamethoxazole (62.50%), chloramphenicol (27.50%), colistin (14.37%), and nitrofurantoin (10.62%) [13]. Furthermore, more than half of the tested strains (52.52%) exhibited multidrug Resistance to multiple antibiotic groups, specifically three or more classes. The disclosure of β-lactamase (ESBL) producing *E. coli* among the isolates further complicates therapeutic management [14]. In this research, we are using cardamom (*Amomum compactum*) and curcumin from turmeric rhizome as substitutes for antibiotics.

Cardamom is one of the species that produces black cardamom and belongs to the genus *Amomum*. In Indonesia, cardamom is Commonly utilized in cooking as well as in traditional medicine for treating conditions such as nausea, alcohol intoxication, tuberculosis, and sore throat [15,16]. The seeds of *A. compactum* contain 60–80% volatile compounds, primarily cineole, which is effective at room temperature and commonly used in aromatherapy and respiratory therapies, including treatments for influenza, nausea, asthma, bronchitis, and other respiratory infections [17]. The antimicrobial mechanism of cineole involves its interaction with transmembrane proteins (porins) located on the outer membrane of bacterial cells. By disrupting the integrity of porins, cineole increases membrane permeability, leading to the leakage of cellular contents, reduced nutrient uptake, and ultimately the inhibition of bacterial growth. The methanol extract of *A. compactum* seeds demonstrated antimicrobial activity in vitro. Using the disk diffusion method, it produced an inhibition zone of 15.15 ± 1.34 mm against *Staphylococcus aureus* at a concentration of 3200 μg/mL, and 14.00 ± 2.54 mm against *Escherichia coli* at 800 μg/mL [18]. Studies have further confirmed that *Amomum compactum* extract possesses antibacterial activity effective against both Gram-positive bacteria—such as *Lactobacillus acidophilus*, *Staphylococcus* spp., and *Streptococcus pyogenes*—and Gram-negative bacteria, including *Salmonella typhimurium* and *E. coli* [19].

Curcumin is a derivative of active polyphenolic herbal substances also referred to as difeuloylmethane [$C_{21}H_{2o}O_{6}$] it is a hydrophobic phytopolyphenol compound found in turmeric rhizomes (*Curcuma* spp.) permeate to the Zingiberaceae family, which is popularly found in Asian continent. Curcumin is the active constituent of turmeric, which is yellow [20]. This substance consists of roughly 6.3% protein, 5–10% fats, 3–7% minerals, and 6–13% moisture [21]. Curcuminoids, though comprising only 3–5% of turmeric’s total composition, encompass over 50 structurally related compounds. Among these, the most prominent are curcumin, demethoxycurcumin, and bisdemethoxycurcumin [22]. Curcumin is frequently used in combination with antibiotics, and this synergy has been authenticate to enhance antibacterial efficacy compared to curcumin administered alone [23]. Gram-negative bacteria similarly like *E. coli* are more sensitive to the combination of curcumin with other bacteriostatic agents.

## 2. Materials and Methods

This study falls within the scope of veterinary health sciences, particularly in the fields of pathology, pharmacology, and veterinary physiology. A descriptive quantitative approach was employed using a cross-sectional experimental design study that involved broiler chicken (*Gallus gallus*) lung tissue as the biological material. This study employed a post-test control group design structured in a multi-group format, comprising eight groups in total: one positive control, one negative control, and six treatment groups.

A total of 32 histopathological slides containing lung tissue from 3-week-old broiler chickens (*Gallus gallus*) were analyzed. These chickens had previously undergone experimental treatment. All chickens were kept under uniform management conditions throughout the 35-day study period. The brooding temperature was initially set at 33 °C during the first week and subsequently reduced by 2 °C each week until reaching 24 °C. Throughout the observation period, feed and drinking water were supplied ad libitum. This study used a Completely Randomized Design (CRD) method consisting of five treatment groups with four replications, each replication containing seven broiler chickens [24]. *E. coli* suspension was administered intraperitoneally into the subjects, and the cardamom extract and curcumin were administered orally.

After the treatment, all subjects were sacrificed. All samples were collected from the subject’s internal organs and blood. Sample grouping was based on the treatment protocols applied in the earlier experimental phase. In this study, the subjects were allocated into four treatment groups to evaluate the effects of Javanese cardamom extract and curcumin at different concentrations on broiler chickens, as outlined in Table 1.

Histomorphometry is a measurement method performed on cells and tissues. Histomorphometry is performed on broiler chicken lung tissue, namely, measuring the area of the parabronchi segment and measuring the area of pathological lesions seen in the preparation. The application of ImageJ software in histomorphometric analysis is intended to facilitate straightforward and standardized measurements, thereby ensuring accuracy and consistency of data across different observers. The use of ImageJ for histological analysis enables both descriptive and quantitative evaluation with greater tenacity and improved accuracy, minimizing variability caused by subjective differences among researchers [25,26]. Incorporating both qualitative and quantitative approaches is crucial to account for possible subjectivity in measurement [27].

## 3. Results

### 3.1. Histopathological Result

We select four histopathological lesions as parameters.

1. Hemorrhage in the lungs of chickens is characterized by the extravasation of blood cells from blood vessels into surrounding tissues. This occurs due to damage to the vessel walls and increased vascular permeability.
2. Inflammatory cell infiltration occurs when immune cells, including neutrophils and lymphocytes, accumulate in areas of infection or inflammation. This reflects the host’s immune reaction to tissue injury or microbial infiltration.
3. Pulmonary edema refers to the accumulation of fluid within lung tissue, which emanate by various aspect such as transportation stress or infection. In stressed broiler chickens, edema can result from elevated vascular pressure, leading to fluid leakage into the interstitial spaces and alveoli.
4. Pulmonary fibrosis involves the formation of scar tissue during the healing process following tissue injury. In chickens, fibrosis may develop as a response to chronic infection or repeated inflammation. This condition is marked with deposition of collagen and other extracellular matrix constituents, resulting in reduced lung elasticity and impaired respiratory function.

**Figure 1:**
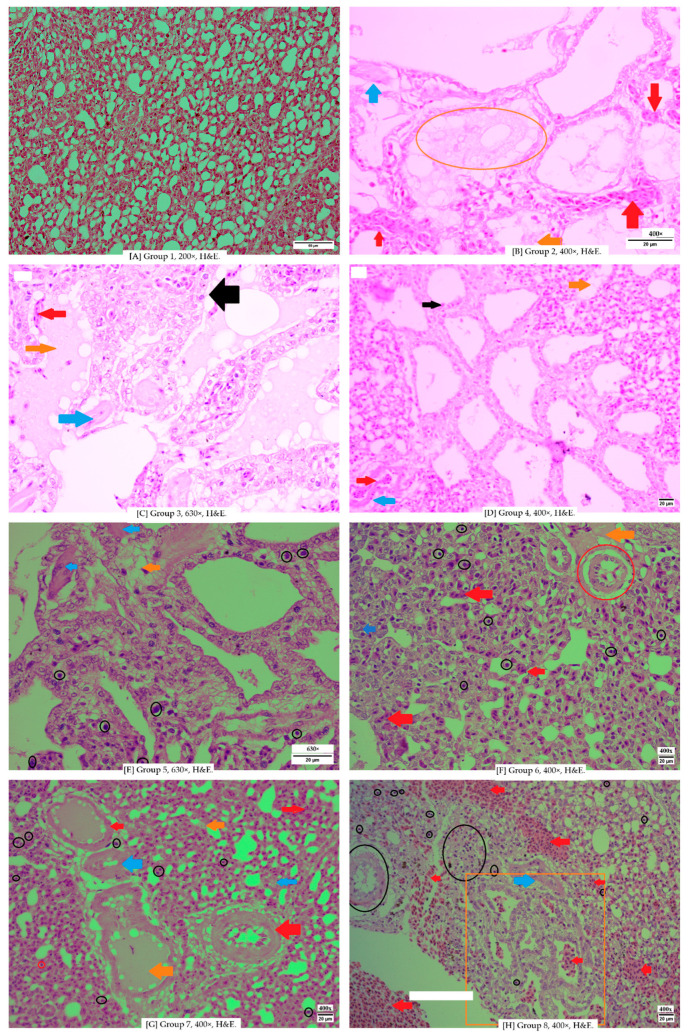
(**A**) is group 1 shows a normal lung from a control negative showing normal lung without damage tissue and cells. (**B**) Group 2 showing parabronchi of pneumonic chicken lungs infected with *Escherichia coli*. (**C**), Group 3 is showing lungs with parabronchi with edema in the cavity, damaged septa, and fibrosis on the septa. (**D**) Group 4 showing parabronchi with good integrity of tissue and septa. (**E**) is group 5, showing lung parabronchi with several heterophils and fibrosis on the septa. (**F**) Group 6 is showing an inflamed pneumocyte, vasculitis on the artery, and thinning of veins. (**G**) is group 7 inflamed pneumocyte, vasculitis on the artery, and thinning of the veins. (**H**) is group 8, showing good integrity of parabronchi, lots of hemorrhage in the side area of parabronchi, the red blood cells in the cavity do not count due to possibly postmortem blood after sacrifice. All the figures showing histopathological pictures of each group and each lesion parameter shown by Different colored arrows indicate specific histopathological features: edema and inflammation of pneumocyte cells (orange arrows/squares/circles), vascular congestion (red solid arrows/hollow squares/hollow circles), hemorrhage (red arrow), and inflammatory cell infiltration as shown in the left image (black arrows and black hollow circles, fibrosis showed with blue solid arrows).

Figure 1 shows a comparison of chicken lung parabronchi. Parabronchi facilitate better and faster air exchange at the blood-gas barrier and better heat release from the body through the lungs. Because the air is more continuously moving through the parabronchi. Group 1 (Figure 1A) is a normal lung from a control negative, Group 2 (Figure 1B) is a pneumonic lung infected with *Escherichia coli,* showing loss of parabronchial septa integrity, thinning of blood vessels, and edema. Group 7 (Figure 1G) and Group 8 (Figure 1H) show 2 different results. Group 7 showing vasculitis, an inflamed pneumocyte, and edema inside the vessel. Group 8 showing normal parabronchial integrity, but much hemorrhage in parenchyma tissue.

### 3.2. Data Analysis

Hypothesis analysis processing using a one-way ANOVA test on both histomorphometric measurement parameters. The results of the measurement data test of the area of the fraction in the field of view of the lungs with a magnification of 400×, a value of *p* = 0.922 [*p* < 0.05]. The findings indicate that no statistically significant differences exist among the groups in the treatment of administering 2 different combinations of doses of Javanese cardamom and turmeric rhizome, and 2 different doses of Javanese Cardamom extract only to the lungs of broiler chickens, which were induced by colibacillosis.

**Figure 2:**
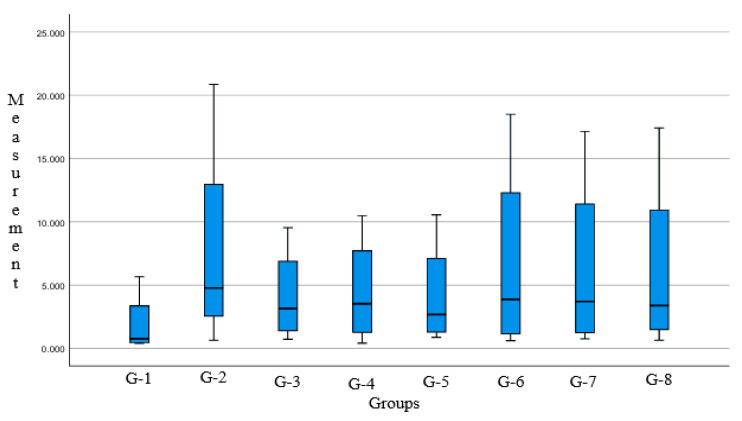
Box-plot diagram area fraction percentage of each group; in terms of statistical difference, the treatment group didn’t show much difference from the positive control group.

**Figure 3:**
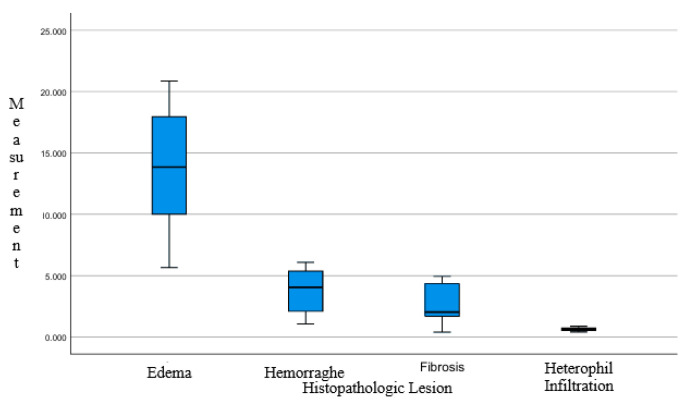
Box-plot diagram percentage of each lesion across the group and samples, showing that edema is a more common lesion that appeared in the groups overall.

The average area fraction of each parameter histomorphometric measurement data of chicken lungs, shown in Table 2, Assessment of data distribution and variance homogeneity was assisted using the Shapiro–Wilk test and Levene’s test, respectively [28]. Statistical analysis result on the data was conducted with histomorphometric measurements in two control groups and six treatment groups. The *p*-value > 0.05 was obtained to validate that the observation data was distributed with normal and homogeneous variance. Data with the characteristics of histomorphometric measurement data of chicken lungs were reviewed for data distribution and homogeneity of variance, and then analyzed using one-way ANOVA. Statistical data processing with IBM SPSS v.27.0 software found that the combination of Javanese cardamom (*Ammomum Compactum*) and turmeric (*Curcuma longa*) had no significant difference in all groups analyzed with one-way Anova, with a significance value [*p* = 0.984]. We can conclude that, based on quantitative measurement and statistical analysis, the performance of Javanese cardamom and turmeric rhizome extract is close to the antibiotics used as comparative control. Group and parameter box-plot graphs are shown in Figure 2 and Figure 3.

## 4. Discussion

### 4.1. E. coli Infection

There are many types of respiratory diseases in the poultry world, including infectious bronchitis, infectious laryngotracheitis, and colibacillosis, which could affect the pulmonary system of avians. Colibacillosis is divided into some species, some of which are known as Enterotoxin *E. coli* (ETEC) or Avian Pathogenic *E. coli* (APEC). Changes in lung tissue conditions, including septal integrity, vascular capillary status, and pneumocyte function, are influenced by pathogenic infection. Infection by Avian Pathogenic *Escherichia coli* (APEC) involves multiple factors of virulence and pathogenic mechanisms that contribute to colibacillosis in poultry. These factors include toxins, iron acquisition systems, invasiveness, protective proteins, and adhesins. The pathogenic mechanisms include adhesion and invasion of host cells, survival within phagocytes (e.g., macrophages), tissue colonization, perseverance in the bloodstream, bacterial replication, cell breakdowns and tissue damage, acquisition of essential metals from body fluids, defiance to oxidative and environmental stress, evasion of serum bactericidal activity, motility, and biofilm formation [29].

APEC infection damages endothelial and epithelial cells in the lungs. Pulmonary epithelial cells serve as primary cellular targets that initiate innate immune responses through the cognizant of pathogen-associated molecular patterns (PAMPs) and damage-associated molecular patterns (DAMPs). Several studies have described the adhesion characteristics of APEC to primary cultures of type II pneumocytes isolated from chickens, which demonstrate high levels of bacterial attachment, leading to epithelial cell damage and the loss of microvilli [21]. This process ultimately contributes to the development of hemorrhage.

**Figure 4:**
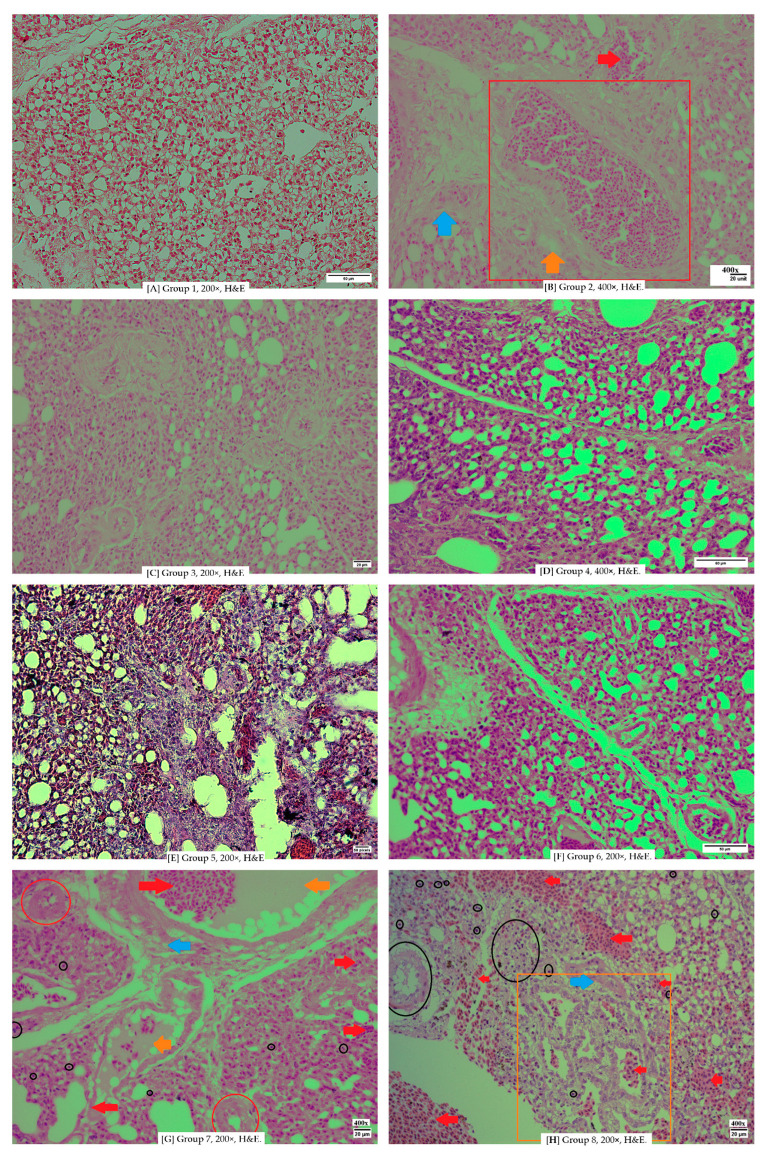
(**A**) is group 1, which shows a normal lung from a control negative, showing a normal lung without damaged tissue and cells. (**B**) is group 2 showing parabronchi of pneumonic chicken lungs infected with *Escherichia coli*, (**C**) is group 3, which shows inflammation in the paranchyme tissue and vasculitis in the vascular. (**D**) Group 4 shows still exhibit intact blood vessels with clearly defined structures. The vascular boundaries remain visible, resembling the connective tissue of the tunica adventitia. (**E**) Group 5 shows a lung tissue that has had fibrosis on parabronchial septa, but didn’t have as much damage to pneumocytes. (**F**) Group 6 shows thinning of the vascular tissue, with visible damage to some vascular walls. This endothelial wall damage is a result of *Escherichia coli* infection, (**G**) is group 7 inflamed pneumocyte, vasculitis on the artery and thinning of veins, (**H**) is group 8 show good integrity parabronchi, lots of hemorrhage on side area of parabronchi, the red blood cell on the cavity doesn’t count due to possibly postmortem bloodafter sacrifice.

In the comparative images above, differences in vascular conditions are observed. The negative control group, as well as Groups 3 (Figure 4C) and 4 (Figure 4D), still exhibit intact blood vessels with clearly defined structures. The vascular boundaries remain visible, resembling the connective tissue of the tunica adventitia. In contrast, Groups 5 (Figure 4E) and Group 6 (Figure 4F) show thinning of the vascular tissue, with visible damage to some vascular walls. This endothelial wall damage is a result of *Escherichia coli* infection.

Infection with *Escherichia coli* activates the host immune response via pathogen-associated molecular patterns (PAMPs), which are perceived by pattern recognition receptors (PRRs). This interaction initiates an inflammatory response characterized by the release of cytokines and chemokines by host cells [30]. Immune responses, particularly those involving heterophils, can generate oxidative compounds—reactive oxygen species (ROS)—such as superoxide anion (O_2_^−^) and hydrogen peroxide (H_2_O_2_), albeit in limited quantities. Upon bacterial invasion of tissue, an acute inflammatory response is triggered, characterized by elevated levels of acute-phase proteins, interleukin-6 (IL-6), cytokine-1, and tumor necrosis factor (TNF), indicating the onset of early pathological changes [31].

**Figure 5:**
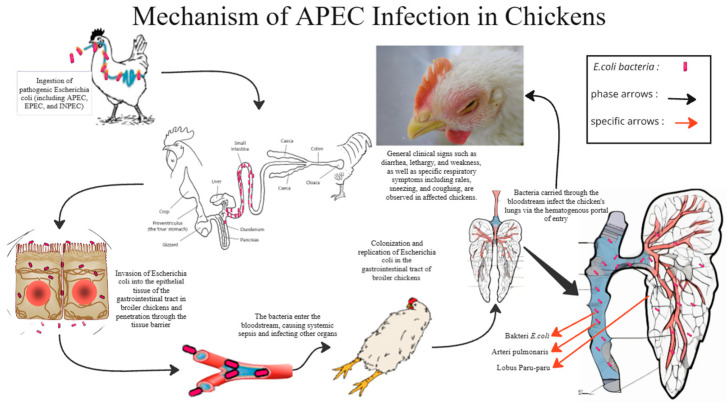
Illustration of *E. coli* infection on a chicken.

Figure 5 illustrates how APEC infects a chicken through the intestine into blood vessels and causing sepsis. Pathogenic infections do not solely trigger immune and inflammatory responses. Other contributing factors include stress, toxicity, and various physiological disturbances. Oxidative stress that exceeds the cellular physiological threshold can disrupt the integrity and function of pulmonary cell membranes, thereby negatively affecting respiratory efficiency and immune system stability. From a pathological perspective, the buildup of reactive oxygen species (ROS) under such conditions can trigger inflammatory pathways and apoptotic processes, thereby contributing to the progression and severity of pulmonary tissue damage [32]. The Javanese cardamom extract and curcumin were administered orally, entered the bloodstream through the mesenteric vein into the abdominal vein, and took a long time to reach the chicken lungs. Then, how does the 1,8-cineol from Javanese cardamom work?

### 4.2. Javanese Cardamom Extract

**Figure 6:**
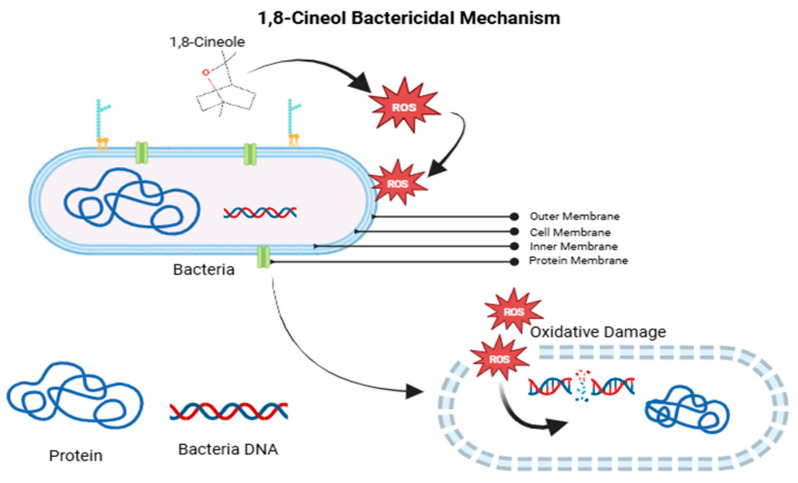
1,8-cineole bactericidal properties affect the ROS-LPS mechanism.

1,8-Cineole exerts its antibacterial effect against *Escherichia coli* by inducing the production of reactive oxygen species (ROS), which lead to oxidative stress and jeopardize the integrity of the bacterial membrane. This results in the disruption of lipopolysaccharides (LPS) and subsequent leakage of intracellular contents.. It also inhibits quorum sensing by suppressing the *luxS* gene, reducing biofilm formation and bacterial virulence. Additionally, 1,8-cineole enhances the effectiveness of antibiotics by lowering the dosage needed to kill the bacteria [33]. These combined actions make 1,8-cineole a powerful agent against *E. coli*, especially in cases of antibiotic resistance. The 1,8-cineole LPS-ROS mechanism is shown in Figure 6.

The negatively charged surface of *Escherichia coli* is primarily due to the high content of lipopolysaccharides (LPSs) in the bacterial outer membrane. 1,8-cineole increases the overall surface energy and leads to the disruption or loss of LPSs. The bactericidal activity of 1,8-cineole is mediated through the generation of reactive oxygen species (ROS), which devotes to the bactericidal effect. The resulting oxidative stress induces membrane damage by promoting lipopolysaccharide (LPS) peroxidation and intracellular membrane leakage, ultimately leading to bacterial cell death [33]. The mechanism of action of 1,8-cineole on the bacterial LPS membrane is illustrated in Diagram 2. In addition to its bactericidal properties, Java cardamom (*Amomum compactum*) also exhibits anti-quorum sensing (QS) activity against *E. coli*. 1,8-cineole interferes with the bacterial quorum-sensing system, disrupting intercellular communication and potentially inhibiting virulence factor expression.

**Figure 7:**
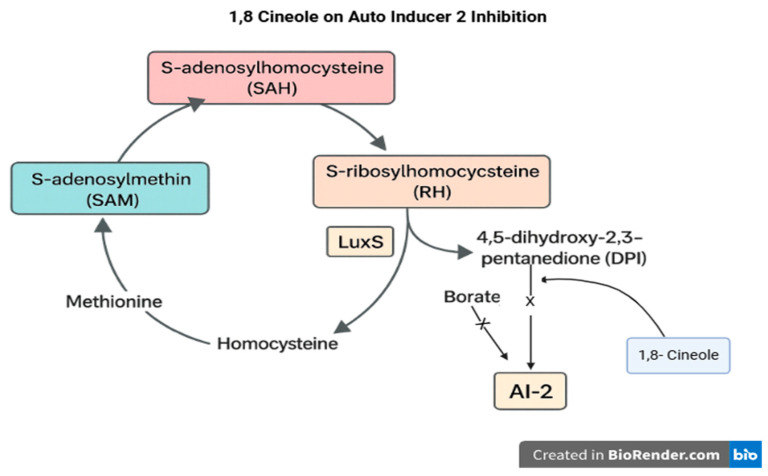
1,8-cineole inhibits the AutoInducer-2 (AI-2) synthesis mechanism. The letter X in the middle of the arrows means the reaction didn’t occur or was inhibited.

Quorum sensing (QS) is a key signaling communication method within bacterial flora [34]. QS involves intercellular communication between bacterial cells straight to the assembly and detection of signaling molecules known as autoinducers (AIs). In *Escherichia coli*, biofilm formation is regulated with autoinducer-2 (AI-2), which is synthesized by the LuxS enzyme. LuxS is a component of the activated methyl cycle and may also affect biofilm development through AI-2–independent pathways by modulating bacterial metabolism [35]. Numerous studies have prove that the *luxS* gene significantly influences both biofilm-forming capacity and bacterial pathogenicity [36]. The quorum sensing mechanism, AI-2 production, and the inhibitory intervention of 1,8-cineole are illustrated in Figure 7.

The AI-2 signaling pathway operates through the binding of 4,5-dihydroxy-2,3-pentanedione (DPD), the precursor molecule of AI-2. The antibacterial efficacy of Java cardamom (*Amomum compactum*) extract is attributed to two primary mechanisms: inhibition of quorum sensing and direct bactericidal activity. To enhance its therapeutic potential, it is combined with curcumin, which provides additional anti-inflammatory and antimicrobial properties, thereby facilitating the healing process.

Curcumin exhibits potent antioxidant properties and serves as an effective free radical scavenger within the body. It also enhances the synthesis of the endogenous antioxidant glutathione (GSH), thereby protecting cells and tissues from oxidative damage. In vitro studies and experiments using animal cells have exposed that curcumin enhances superoxide dismutase (SOD) activity and increases glutathione (GSH) levels in both cellular and serum environments. Lee et al. 2010 [36] Curcumin supplementation has been reported to reduce the production of reactive oxygen species (ROS) in lung endothelial cells following γ-irradiation. Its anti-inflammatory effects are principally mediated through the inhibition of free radical activity via the NF-κB, TGF-β, and mitogen-activated protein kinase (MAPK) signaling pathways. Additionally, curcumin exhibits potent antioxidant properties by activating the Nrf2 pathway, which enhances cellular antioxidant defenses through the upregulation of enzymes such as catalase (CAT), superoxide dismutase 1 (SOD1), and glutathione peroxidase 1 (GPX1). It also modulates the function of channel proteins and molecular chaperones, further supporting cellular protection mechanisms [37].

**Figure 8:**
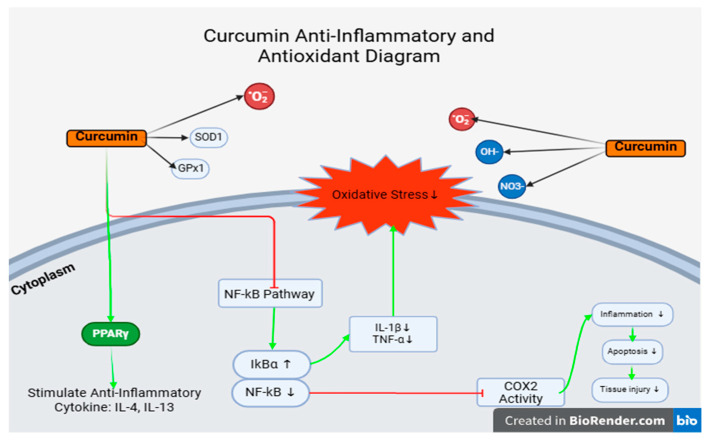
Anti-inflammation and antioxidant mechanisms in curcumin. Green arrows show curcumin affects directly, red line shows affects indirectly, black arrows show curcumin catches ROS.

Curcumin has also been recognized as a potent anti-drug resistance agent. It exhibits a unique ability to withhold the overexpression of P-glycoprotein and its mRNA induced by Adriamycin, thereby increasing intracellular drug accumulation [38]. These mechanisms are illustrated in Figure 8.

Based on the discussion that was presented, it is proven that 1,8-cineol from Javanese cardamom extract has quite good antibacterial properties in inhibiting bacterial quorum sensing (quorum sensing), and *C. longa* extract (*Curcumin*) has antibacterial function against various types of bacteria, by being an amphipathic and lipophilic molecule. Curcumin is incorporated into the liposome bilayer, increasing membrane permeability. The results of statistical data processing with IBM SPSS V.27 found that the combination of Javanese cardamom (*Ammonum Compactum*) and turmeric (*Curcuma longa*) had no significant difference in all groups analyzed with one-way Anova with a significance value [*p* = 0.922].

In this study, we compare the mix of rhizome and Curcuma to one of the common antibiotics that are used as an anti-colibacillosis, namely, ciprofloxacin, which has a DNA-Gyrase effect on the bacteria. We have found no significant difference [*p* > 0.05] between the groups in this study. This represents the antibacterial effect as promised in Curcuma, and the rhizome could be equal to ciprofloxacin that was used in this study; it could be safer because the nature of the Curcuma and rhizome combination is from plant/herbs.

We compare the severity of each group, namely, Group 3, which received an induction of 0.5 mL of *E. coli* bacterial suspension with an infection dose of 10^8^ CFU/mL/head, 0.06 mL/Kg BW of Javanese cardamom extract, and 400 mg of curcumin. We’re found minimal area inflammation, and pittance vascular damage leading to hemorrhage, more heterophils that appeared in every field of view, closely over medium-sized vascular diameter.

Group 4 received an induction of 0.5 mL of *E. coli* bacterial suspension with an infection dose of 10^8^ CFU/mL, 0.1 mL/Kg BW of Javanese cardamom extract, and 400 mg of curcumin, showing slightly more edema compared to group 4 in several field of view, less hemorrhage lesions, and the lowest average of heterophil infiltration from the entire group.

Group 5 received 0.5 mL of *E. coli* bacterial suspension with an infection dose of 10^8^ CFU/mL/, 0.06 mL/Kg BW of Javanese cardamom extract. Histopathological findings in group 5 didn’t show much difference from group 4, but the fibrosis was quite low.

Group 6 with 0.5 mL of *E. coli* bacterial suspension with an infection dose of 10^8^ CFU/mL and 0.1 mL/Kg BW of Javanese cardamom extract showed higher hemorrhage, as much as the control positive group, much higher edema, low fibrosis, and average heterophil infiltration. The parabronchi in group 6 were more damaged on the septa.

Group 7 received an induction of 0.5 mL of *E. coli* bacterial suspension with an infection dose of 10^8^ CFU/mL/head orally + 400 mg/kg feed/day of curcumin. It’s also had higher edema and hemorrhage, and an amount of heterophil that similar to group 6.

The histopathological findings indicate that Javanese cardamom extract reduces bacterial proliferation in the bloodstream by inhibiting bacterial quorum sensing. Additionally, it generate the production of reactive oxygen species (ROS), which in high concentrations can trigger inflammation, damage proteins, DNA, lipids, and lipid membranes, and also induce apoptosis. This oxidative stress situation is an advantage to eliminate bacteria/pathogens, but brings out disadvantages if it occurs for a long time or in a wider area. Curcumin is an answer for these disadvantages by scavenging the ROS and giving immunomodulation properties by reducing inflammation.

After comparison of findings, Javanese cardamom and turmeric rhizomes extract are capable of the same performance as ciprofloxacin in a dose of 0.06 mL of cardamom extract only or combined with 400 mg of curcumin.

A histomorphometric study presents several limitations, particularly in the areas of sample preparation, implementation, and result interpretation. The researchers have identified the following limitations to inform readers and provide context for future investigations:

1. The lesion grading lacked standardization and clear criteria for severity, leading to its exclusion as a variable in the data analysis.
2. The histological findings in lung tissue may not accurately represent or correlate with the degree of damage in other organs. Therefore, pulmonary tissue damage should not be assumed to reflect systemic pathological conditions.

This study has certain limitations in its implementation. Therefore, the researchers propose the following suggestions and inputs for future researchers who may wish to continue or adapt this methodology. These also represent potential research gaps that can be explored in subsequent studies:

1. The examination of other organs such as the liver, kidneys, and heart is recommended to obtain a more comprehensive understanding of systemic pathological effects.
2. The establishment of a standardized grading system for tissue damage based on lesion severity would enhance the accuracy and consistency of histopathological evaluations.
3. Alternative diagnostic techniques may be employed, such as Ziehl–Neelsen staining for bacterial visualization in tissues or immunohistochemistry (IHC) to detect specific antibody responses at the tissue level.

## 5. Conclusions

All findings, results, and discussions of this study can be concluded by looking at points that can answer research questions based on the research objectives as follows:

1. There’s no significant differences were observed in the histomorphometric measurements of broiler chicken lungs treated with a combination of Java cardamom extract and turmeric extract. The range of administered doses did not produce statistically significant effects. One-way ANOVA analysis showed a non-significant p-value [*p* = 0.922; *p* > 0.05] between intervention groups and the control group. Based on histopathological evaluations, there were no significant differences in lesion severity or tissue integrity across all groups. Common findings such as pneumocyte inflammation, damage to parabronchial structures (e.g., necrosis, inflammation, and cellular fluid accumulation), and hemorrhage were observed consistently in all microscopic fields across all groups. The infiltration of immune cells was present but not markedly significant in any field of view.
2. Improvement in pathological conditions was noted in Group 3 (G3) and Group 6 (G6). Group 3 received a dose of 0.06 mL/kg BW of Java cardamom extract combined with 400 mg of curcumin extract, while Group 6 received 0.06 mL/kg BW of Java cardamom extract only.
3. In practical applications, these results may benefit poultry farms. However, certain concerns should be considered, particularly regarding the concentration of cardamom extract. The compound 1,8-cineole can cause a burning sensation in the digestive tract, potentially discouraging feed consumption due to swallowing difficulties. Excessive concentration may also lead to lesions in the upper digestive tract.

## Figures and Tables

**Table 1:** Subject Grouping for the in vivo study.

Group	Treatment [24]
1. Negative Control	0.5 mL of Isotonic saline solution orally daily.
2. Positive Control	0.5 mL of *E. coli* mixed suspension with an infection dose of $10^{8}$ CFU/mL/BW, then received 0.5 mL of Isotonic saline solution orally daily
3.	0.5 mL of *E. coli* bacterial suspension with an infection dose of $10^{8}$ CFU/mL/BW + a mixture of 0.06 mL/kg BW of cardamom essential oil and 400 mg/kg feed/day of curcumin orally daily
4.	0.5 mL of *E. coli* bacterial suspension with an infection dose of $10^{8}$ CFU/mL/BW + a mixture of 1 mL/kg BW of cardamom essential oil and 400 mg/kg feed/day of curcumin orally every day
5.	0.5 mL of *E. coli* bacterial suspension with an infection dose of $10^{8}$ CFU/mL/BW + 0.06 mL/kg BW of cardamom essential oil only orally every day
6.	0.5 mL of *E. coli* bacterial suspension with an infectious dose of $10^{8}$ CFU/mL/BW + 1 mL/kg BW of cardamom essential oil orally every day
7.	0.5 mL of *E. coli* bacterial suspension with an infectious dose of $10^{8}$ CFU/mL/BW + 400 mg/kg feed/day of curcumin orally every day
8.	0.5 mL of *E. coli* bacterial suspension with an infectious dose of $10^{8}$ CFU/mL/BW + ciprofloxacin antibiotic 1 g/2 L of drinking water

**Table 2:** Average measurements from each lesion by each group.

Group	Histopathological Lesions [% Area]			
	Edema	Hemorrhage	Fibrosis	Heterophil Infiltration
G-1	5.662	1.060	0.394	0.469
G-2	20.852	5.073	4.457	0.650
G-3	9.539	2.062	4.211	0.721
G-4	10.483	2.121	4.930	0.407
G-5	10.545	3.652	1.687	0.881
G-6	18.496	6.080	2.132	0.612
G-7	17.145	5.655	1.746	0.751
G-8	17.415	4.440	2.326	0.640

## Data Availability

The primary data supporting the findings of this study are included within the article. Additional data is available from the corresponding author upon reasonable request.

## Abbreviations

APEC	Avian Pathogenic *Escherichia Coli*
SBM	Stored Biological Material
CNF-1	Cytotoxic Necrotizing Factor 1
DPPH	Diphenyl Picryl Hydrazyl Hydrate
DOC	Day-Old Chicken
IFN-γ	Interferon Gamma
IL-1β	Interleukin 1β
IL-4	Interleukin 4
IL-5	Interleukin 5
IL-6	Interleukin 6
IL-10	Interleukin 10
Ig A	Immunoglobulin A
Ig E	Immunoglobulin E
Ig G	Immunoglobulin G
DNA	Deoxyribonucleic Acid
mRNA	Messenger Ribo-Nucleic Acid
OVA	Ovalbumin
BALF	Broncho-Aveolar Lavage Fluid
HO-1	Heme Oxygenase 1
Th-2	T Helper 2
ROS	Reactive Oxygen Species
COX-2	Cyclooxygenase-2
iNOS	Inducible Nitric Oxide Synthase
AMR	Antimicrobial Resistance
LD50	Lethal Dose 50
H&E	Hematoxylin & Eosin
